# Distinct clinical profiles and mutation landscapes of gliomas originating from the neocortex, mesocortex, and cerebellum

**DOI:** 10.1016/j.gendis.2023.02.044

**Published:** 2023-04-05

**Authors:** Ye Cheng, Lei Zhang, Xiaolong Wu, Hiroaki Wakimoto, Haoming Geng, Yukui Wei, Geng Xu, Xinru Xiao, Jie Bai, Yaming Wang, Zeliang Hu, Leiming Wang, Qingtang Lin

**Affiliations:** a Department of Neurosurgery, Xuanwu Hospital, Capital Medical University, Beijing 100053, China; b Brain Tumor Research Center, Massachusetts General Hospital, Department of Neurosurgery, Harvard Medical School, Boston, MA 02134, USA; c Department of Pathology, Xuanwu Hospital, Capital Medical University, Beijing 100053, China

Gliomas originating from anatomically and developmentally distinct brain regions have different clinical outcomes. However, the molecular landscape underlying this difference remains largely unknown. We analyzed key molecular mutations via sequencing and correlated them with clinical characteristics in 180 adult patients with gliomas originating from the neocortex, mesocortex, and cerebellum. Cases of cerebellar origin had significantly longer survival than those of supratentorial origin, consistent with higher rates of mutation in key genes associated with supratentorial gliomas. In high-grade gliomas (HGGs), shorter survival was found in cases of neocortex origin compared to those of mesocortex or cerebellum origin, consistent with a higher rate of mutation in the *hTERT* promotor. In low-grade gliomas (LGGs), cases of supratentorial origin also exhibited higher mutation rates in specific genes. Different driver genes that potentially underlie the progression from LGG to HGG were identified for glioma per origin. In summary, our data shed light on the molecular mechanisms for the different clinical characteristics of gliomas of different origins. These findings highlight the heterogeneity of the molecular landscape of gliomas originating from different brain regions and can provide a critical foundation for targeted therapy against gliomas.

The development of the human brain occurs through the exquisitely timed process of cortical organization and formation of the allocortex, mesocortex, and neocortex. Generally, gliomas of the cerebellar and mesocortical brain regions tend to have a better clinical prognosis than gliomas originating from neocortical brain regions. Gliomas originating from different brain regions may be caused by different mutations that require different therapeutic strategies and may have different prognoses.^1^ Here, we first stratified the glioma cohort by cortical region of origin and investigated the association of tumor origin with parameters such as sex, age, location, mutation status (e.g., *IDH*), and *MGMT* promoter methylation. We determined the involvement or lack of involvement of each brain lobe by examining the enhancement due to tumor tissue on images from T1-weighted sequences with gadolinium or T2 flair. Tumor origins were determined by experienced neurosurgeons (Q.T. Lin and Y. Cheng) using magnetic resonance imaging with the multiplanar reformation, which enabled tumor localization in axial, sagittal, and coronal views. Based on brain topography, gliomas involving the frontal, temporal, parietal, and occipital lobes were assigned to the neocortex group. Gliomas that involved supratentorial non-neocortical regions such as the cingulate, anterioinferoinsular cortex, and insular lobe were assigned to the mesocortex group. Cerebellar gliomas were identified as the third group (Fig. 1A). Gliomas involving both neocortex and mesocortex, diffuse midline gliomas (H3.3), and multiple cerebral gliomas were excluded. Patients with adverse events (eight cases) were also excluded. As a result, a total of 180 glioma cases managed at our institution by standardized treatment^2^ over seven years were enrolled, including 101 gliomas originating from the neocortex, 45 from the mesocortex, and 34 from the cerebellum.

**Figure 1:**
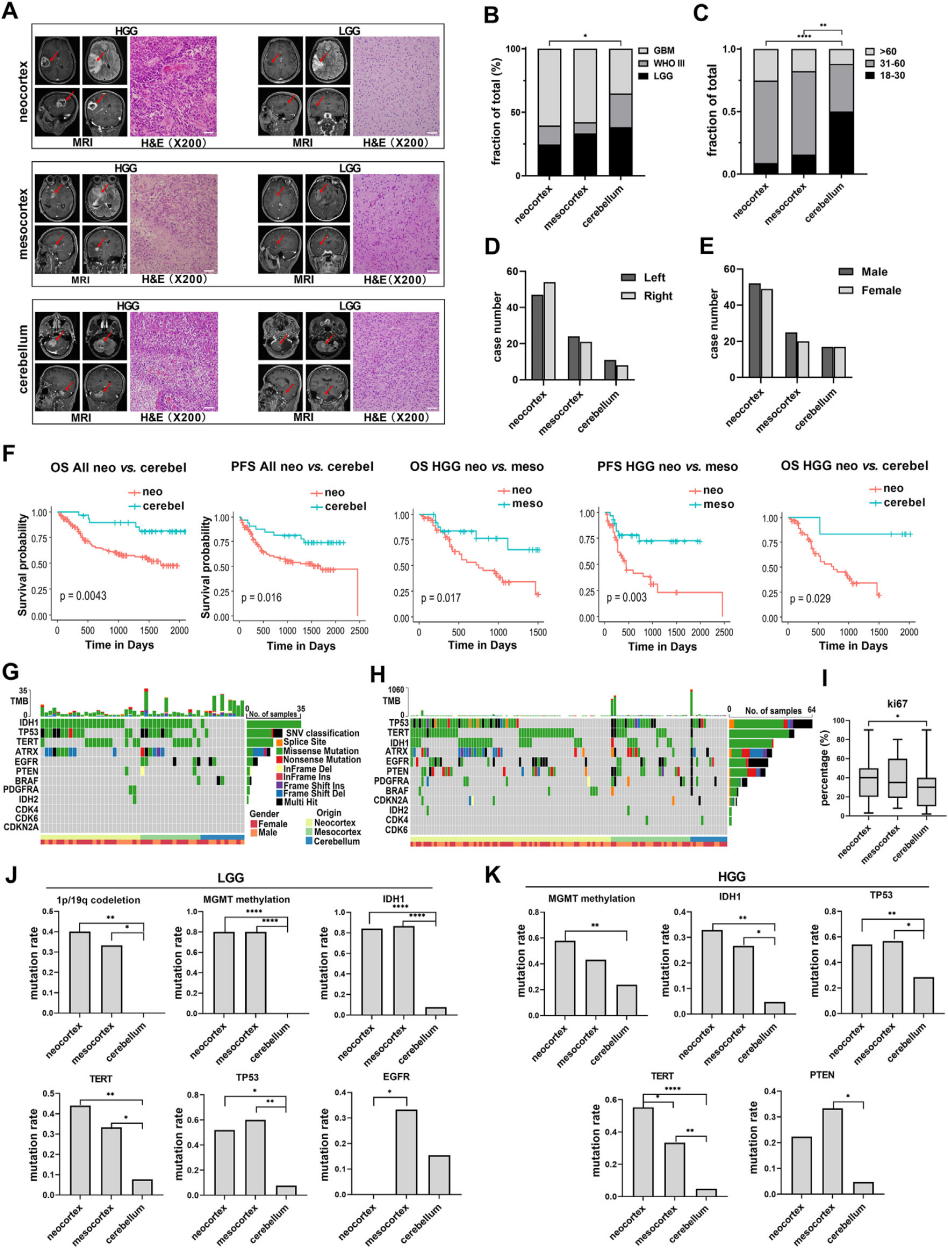
Clinical characteristics and mutation landscapes of gliomas originating from the neocortex, mesocortex, and cerebellum. **(A)** Typical imaging and H&E-stained sections of glioma originating from different cortices. **(B)** Age, **(C)** grade, **(D)** side, and **(E)** sex distribution of gliomas originating from different cortices (chi-square test, ∗*P* < 0.05, ∗∗*P* < 0.01, ∗∗∗∗*P* < 0.0001). **(F)** Comparison of OS and PFS between gliomas of neocortex origins and mesocortex origins, as well as between neocortex origins and cerebellar origins (Kaplan Maier). **(G)** Mutation status of key genes in LGGs. **(H)** Mutation status of key genes in HGGs. **(I)** Ki-67 percentage in gliomas originating from the neocortex, mesocortex, and cerebellum (*t*-test, ∗*P* < 0.05). **(J)** Comparison of mutation rates of key genes in LGGs originating from different cortices. **(K)** Comparison of mutation rates of key genes in HGGs originating from different cortices. (chi-square test; ∗*P* < 0.05, ∗∗*P* < 0.01, ∗∗∗∗*P* < 0.0001). Cerebel, cerebellum; H&E, hematoxylin and eosin staining; HGG, high-grade glioma; LGG, low-grade glioma; meso, mesocortex; MRI, magnetic resonance imaging; neo, neocortex; OS, overall survival; PFS, progression-free survival.

We then investigated the correlation between tumor origin and clinical properties. As a result, the percentages of HGG versus LGG in the neocortex, mesocortex, and cerebellum were 75.25% (76/101) versus 24.75% (25/101), 66.67% (30/45) versus 33.33% (15/45), and 61.76% (21/34) versus 38.24% (13/34), respectively. A significant difference was found in the grade distribution between the neocortex and cerebellum (Fig. 1B). Significant differences were found in age distribution between supratentorial and cerebellar gliomas (*P* < 0.05, chi-square test) (Fig. 1C). No differences in sides and sex were found between gliomas originating from different cortices (Fig. 1D, E). Survival and prognosis of gliomas originating from the neocortex, mesocortex, and cerebellum remained unknown. By analyzing the following-up data of the total 180 patients, we found significant differences in overall survival (OS) and progression-free survival (PFS) between gliomas originating from the neocortex and cerebellum. In HGGs, OS and PFS were significantly different between tumors originating from the neocortex and mesocortex, while the difference in OS was significant between HGGs originating from the neocortex and cerebellum. There was a difference in PFS between LGGs originating from the mesocortex and cerebellum (*P* < 0.01) (Fig. 1F; Fig. S1, 2).

Clinically, gliomas of the mesocortex and cerebellum often exhibit a better clinical prognosis in comparison with gliomas of the neocortex. However, the key molecular landscape underlying this difference remains to be explored. To understand the molecular mechanism under the distinct clinical profiles of glioma originating from different origins, we analyzed key molecular mutations via sequencing and correlated them with the clinical characteristics. Genetically, higher rates of *1p/19q* co-deletion, *MGMT* promoter methylation, as well as *IDH1*, *hTERT* promoter, *ATRX*, and *TP53* mutations were found in LGGs originating from the neocortex and mesocortex than in those originating from the cerebellum (*P* < 0.05, *t*-test) (Fig. 1G, H; Fig. S3). *EGFR* amplification is the key molecular marker that predicts the aggressive clinical course of IDHwt LGGs.^3^ Importantly in our study, *EGFR* mutation was absent in LGGs from the neocortex, and the mutation rate was significantly lower than that in mesocortex LGGs, suggesting *EGFR* amplification in the mesocortex gliomas may be a potential predictor for survival. The Ki-67 index was higher in neocortex HGGs compared with that in cerebellar HGGs (Fig. 1I). Genetically, a higher rate of *MGMT* promoter methylation and a higher prevalence of IDH1 mutation was found in HGGs originating from the neocortex than in those originating from the cerebellum. Higher rates of *hTERT* promoter and *TP53* mutations were found in supratentorial gliomas (both the neocortex and mesocortex) than in the cerebellum. Mesocortex HGGs exhibited significantly longer survival than their neocortex counterparts. Notably, a higher rate of *hTERT* promoter mutation was found in HGGs originating from the neocortex than in HGGs from the mesocortex (Fig. 1I, K; Fig. S4) (*P* < 0.05, *t*-test). *TERT* expression is associated with poor outcomes in most tumors including gliomas. For example, mutations in the *TERT* promoter predict poorer OS in HGGs without *IDH1/2* mutations and serve as an independent prognostic factor for poor outcomes.^4^

Grade progression of glioma-intrinsic genes has been previously reported.^5^ By analyzing the mutation status of both the primary and secondary groups, we further identified several candidate driver genes at two time points (*i.e.*, primary and recurrent gliomas) originating from different cortices. In the neocortex, we found recurrent loss-of-function mutations in *LRP1B*, *FAT1*, *PIK3CA*, *SETD2*, *BRCA2*, *PIK3R1*, and *PTEN. NF2* mutations were found only in the secondary neocortex and cerebellum, which indicates an evolutionary factor, but not in the mesocortex. According to our results, *PTEN* and *FAT1* mutations were confined to recurrent tumors in all samples (Fig. S5). When considering tumor origin, our observational study also found differences in mutations between primary and secondary gliomas originating from the neocortex, mesocortex, and cerebellum, which could be the first analysis of this type. Future studies should focus on statistical evidence and include a larger sample containing both primary and secondary gliomas.

In summary, our findings highlight the heterogeneity of gliomas of different origins, in terms of their clinical characteristics, key molecular mutations, and malignant progression pathways. Clinically, these results expand our understanding of the molecular course and malignant progression of gliomas, especially for gliomas of the mesocortex and cerebellum, long-time neglected entities, and provide an important foundation for future clinical-molecular integrated classification, targeted drug development, and individualized therapeutic strategies for gliomas of distinct origins. Future study is warranted with a larger study sample size and analysis of detailed mechanisms underlying the mutational landscapes.

## Author contributions

Conception and design: Y.C., L.W., and Q.L. Data collection and analysis: Y.C., L.Z., X.W., and H.G. Supervision: Y.W., G.X., X.X., J.B., Y.W., and Z.H. Writing - original draft: Y.C., L.Z., and X.W. Writing - review & editing: Y.C., L.W., Q.L., and H.W. All authors contributed to the manuscript and approved the submitted version.

## Conflict of interests

There is no conflict of interests to declare.

## Funding

This work was funded by the Youth Program of the 10.13039/501100001809National Natural Science Foundation of China (No. 81802485 to Y. Cheng) and the Beijing New-star Plan of Science and Technology (China) (No. Z201100006820148 to Y. Cheng, No. Z201100006820149 to L.M. Wang).

## References

[1] Lopci E., Riva M., Olivari L. (2017). Prognostic value of molecular and imaging biomarkers in patients with supratentorial glioma. Eur J Nucl Med Mol Imag.

[2] Stupp R., Hegi M.E., Mason W.P. (2009). Effects of radiotherapy with concomitant and adjuvant temozolomide versus radiotherapy alone on survival in glioblastoma in a randomised phase III study: 5-year analysis of the EORTC-NCIC trial. Lancet Oncol.

[3] Park Y.W., Ahn S.S., Park C.J. (2020). Diffusion and perfusion MRI may predict EGFR amplification and the TERT promoter mutation status of IDH-wildtype lower-grade gliomas. Eur Radiol.

[4] Aldape K., Zadeh G., Mansouri S. (2015). Glioblastoma: pathology, molecular mechanisms and markers. Acta Neuropathol.

[5] Wang Q., Hu B., Hu X. (2017). Tumor evolution of glioma-intrinsic gene expression subtypes associates with immunological changes in the microenvironment. Cancer Cell.

