# Supplementary material for: Distinct clinical profiles and mutation landscapes of gliomas originating from the neocortex, mesocortex, and cerebellum
Source: Genes Dis. 2023 Apr 5;11(1):53–6. doi: 10.1016/j.gendis.2023.02.044 (PMC10425792; doi:10.1016/j.gendis.2023.02.044)
Supplement: Multimedia component 1 [file mmc1.docx]

**Supplementary figures**


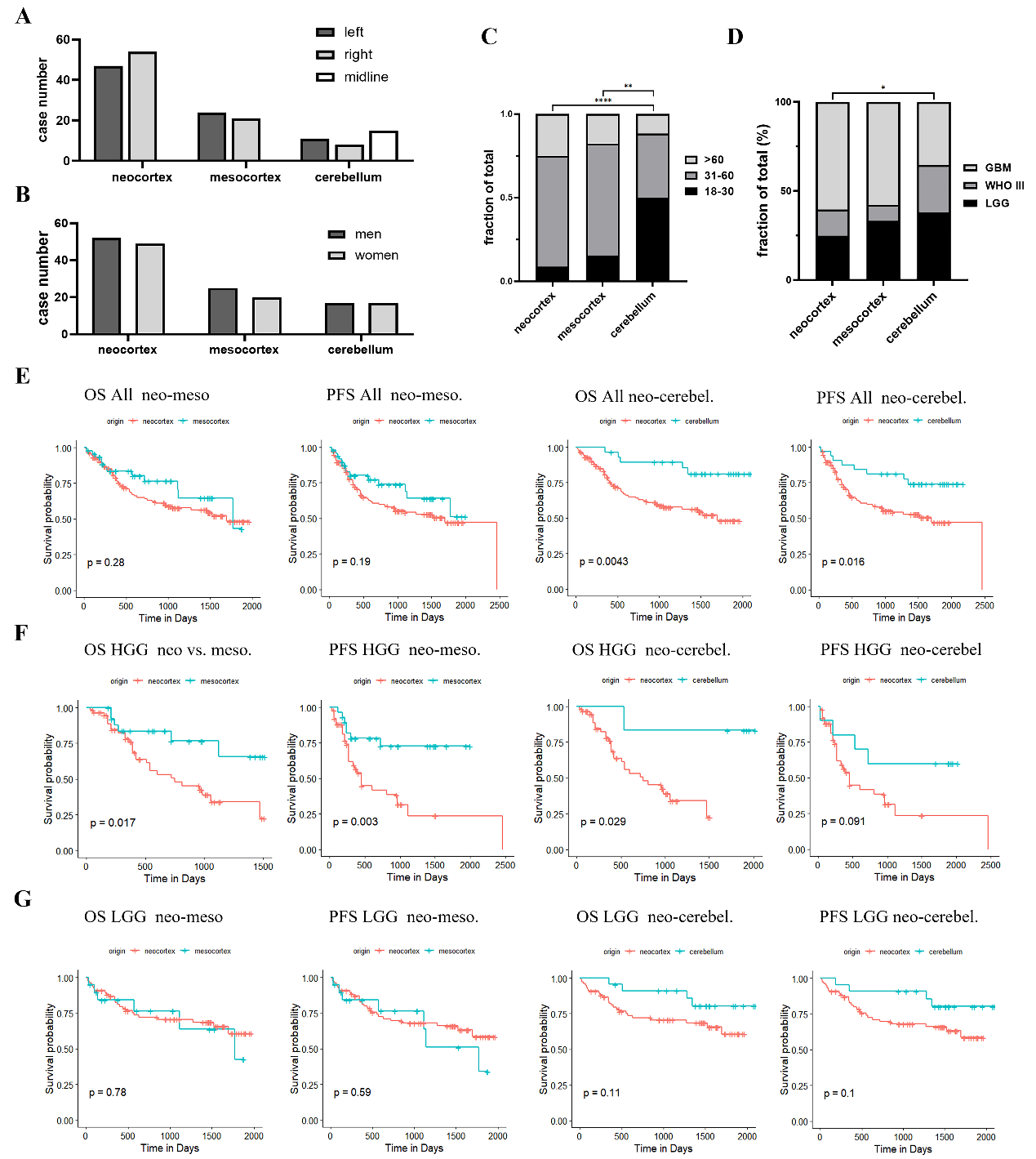
**Fig. S1.** Clinical characteristics, mutation status, and prognostic features of gliomas originating from different cortices. (A) Side distribution. (B) Sex distribution. (C) Grade distribution. (D) Age distribution. (E) Comparison of OS and PFS between gliomas of neocortex and mesocortex origins, as well as between neocortex and cerebellar origins. (F) Comparison of OS and PFS between HGGs of neocortex and mesocortex origins, as well as between neocortex and cerebellar origins. (G) Comparison of OS and PFS between LGGs of neocortex and mesocortex origins, as well as between neocortex and cerebellar origins. (chi-square test; *, p < 0.05; **, p < 0.01; ***, p < 0.001, ****, p < 0.0001).

LGG, low-grade glioma; HGG, high-grade glioma; OS, overall survival; PFS, progression-free survival; neo, neocortex; meso, mesocortex; cerebel, cerebellar; GBM, Glioblastoma multiforme.

**
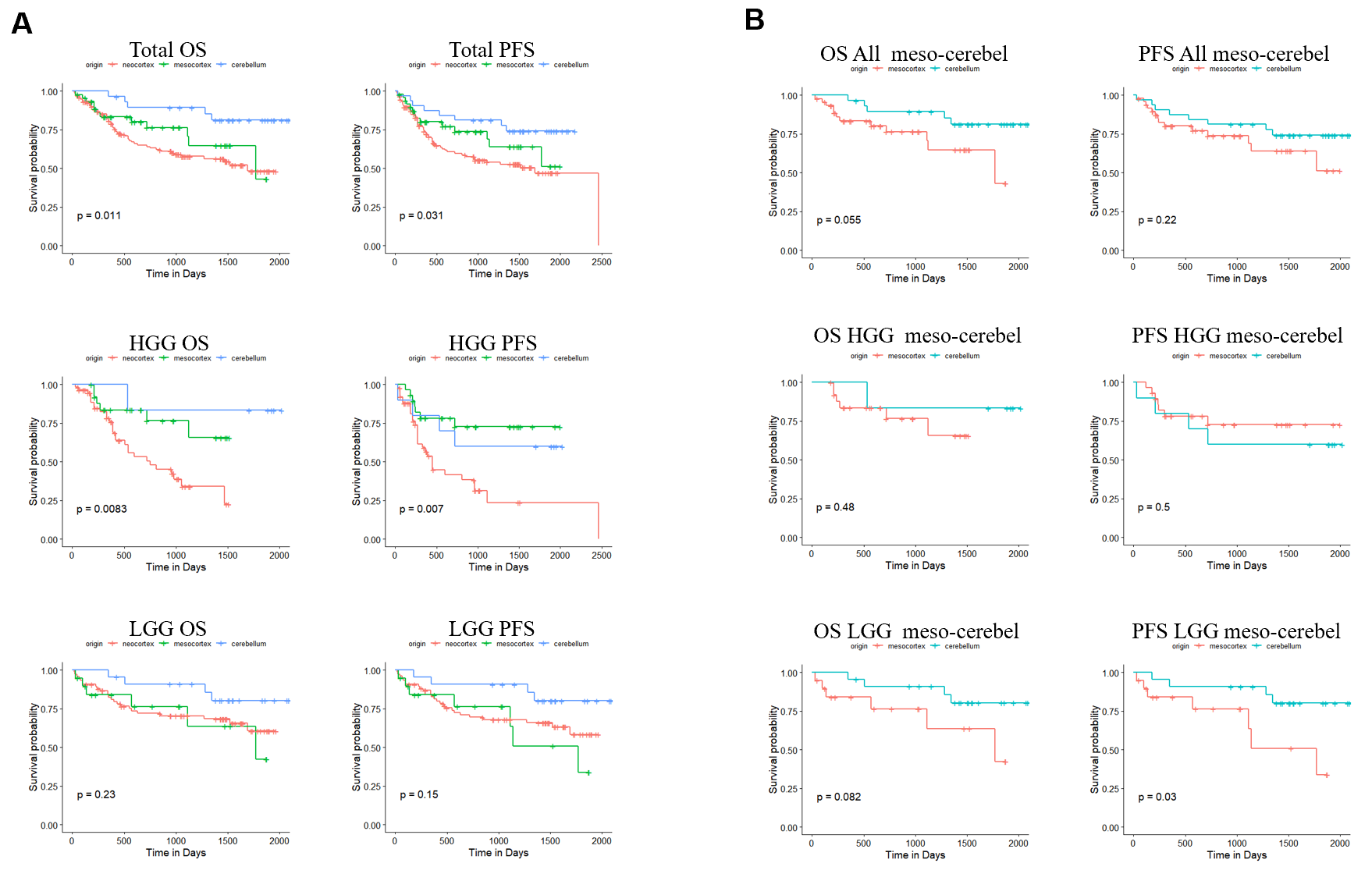
**

**Fig. S2.** (A) Comparison of OS and PFS in all cases. (B) Comparison of OS and PFS between HGGs of mesocortex and cerebellum origins.

HGG, high-grade glioma; OS, overall survival; PFS, progression-free survival


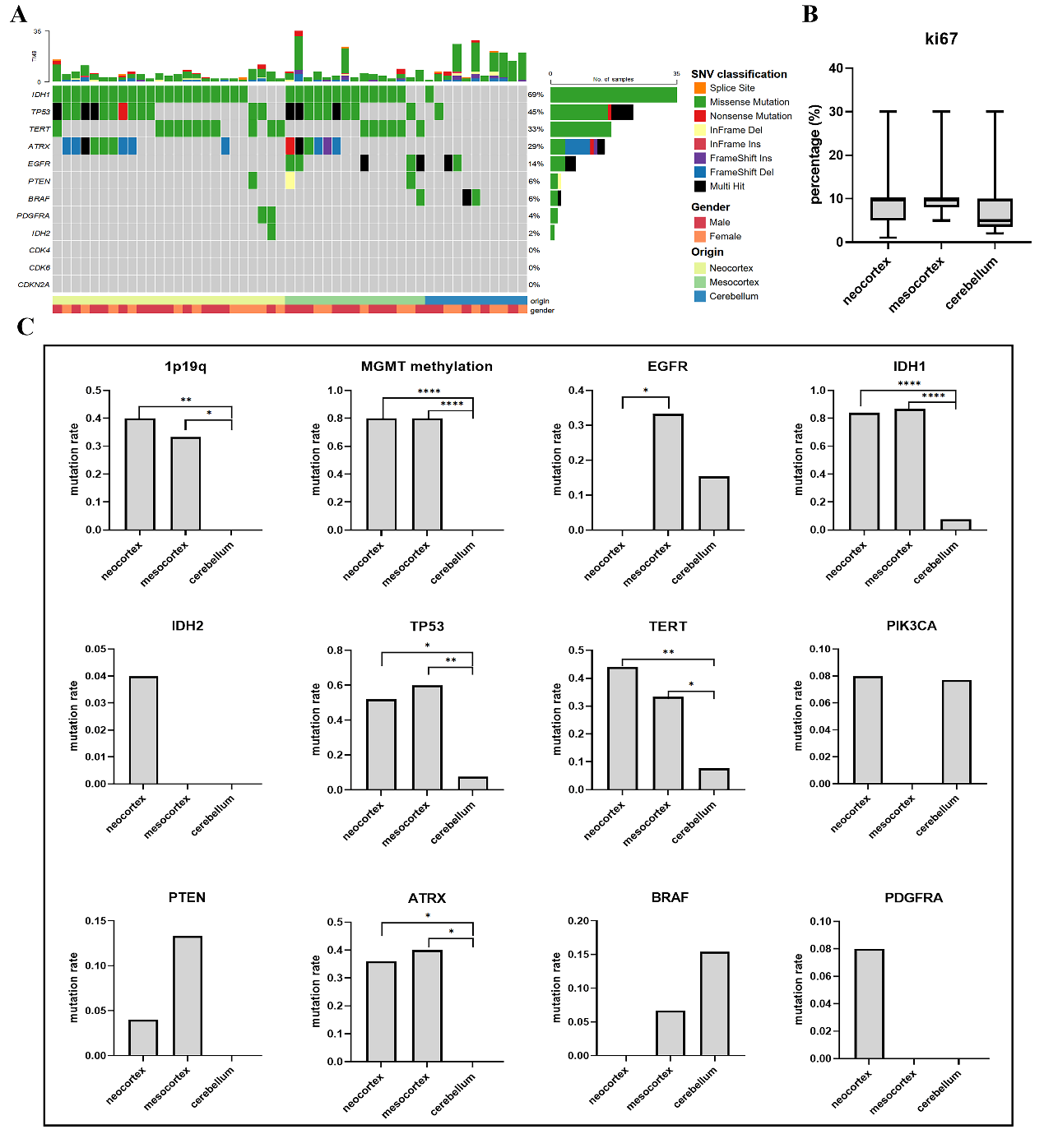


**Fig. S3.** Mutation status of key genes in LGGs originating from different cortices**.** (A) Mutation status of key genes in LGGs. (B) Ki-67 percentage in LGGs originating from the neocortex, mesocortex, and cerebellum (t-test; *, p < 0.05). (C) Comparison of mutation rates of key genes (chi-square test; *, p < 0.05; **, p < 0.01; ****, p < 0.0001).

LGG, low-grade glioma.


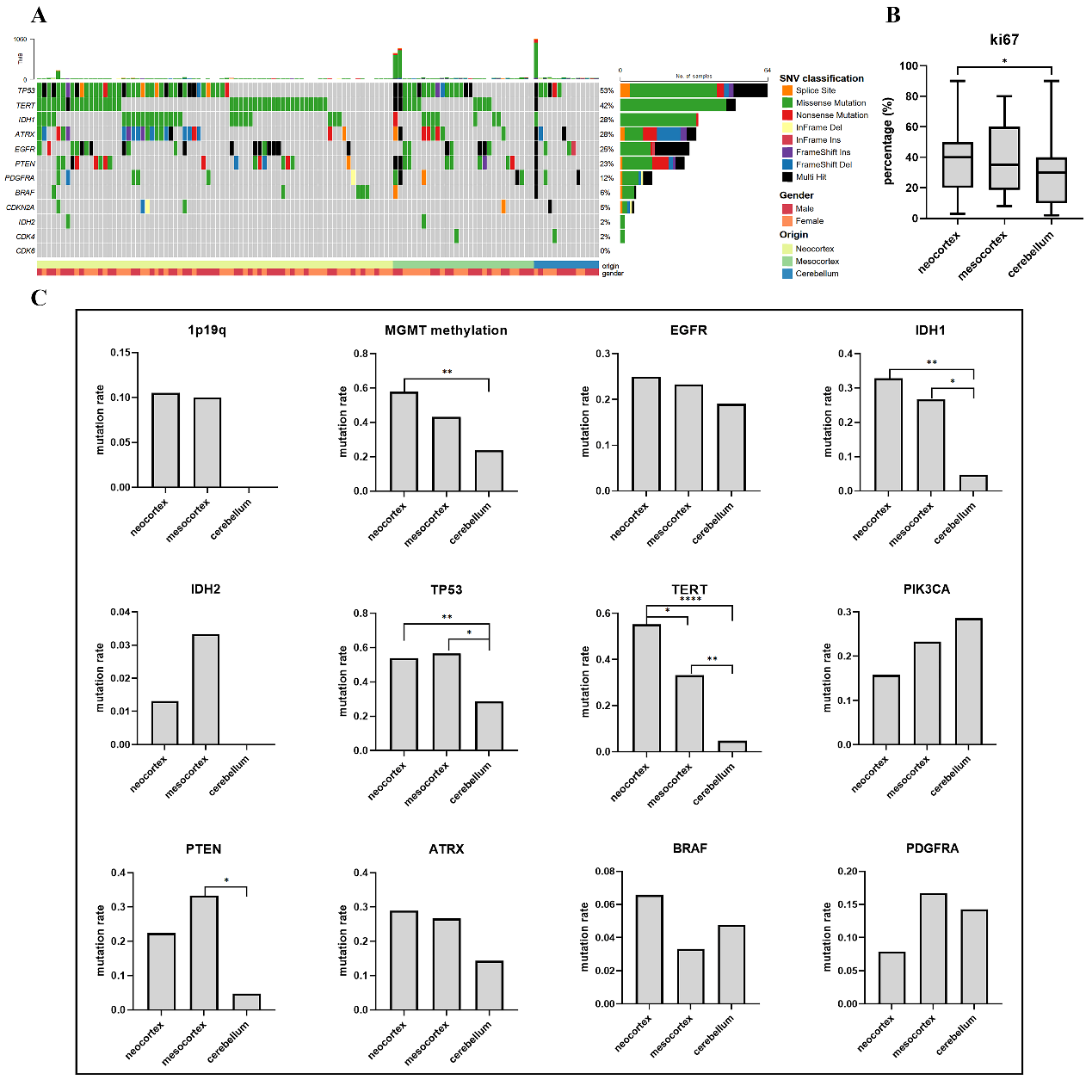


**Fig. S4.** Mutation status of key genes in HGGs originating from different cortices. (A) Mutation status of key genes in HGGs. (B) Ki-67 percentage in gliomas originating from the neocortex, mesocortex, and cerebellum (t test; *, p < 0.05). (C) Comparison of mutation rates of key genes (chi-square test; *, p < 0.05; **, p < 0.01; ****, p < 0.0001).

HGG, high-grade glioma.


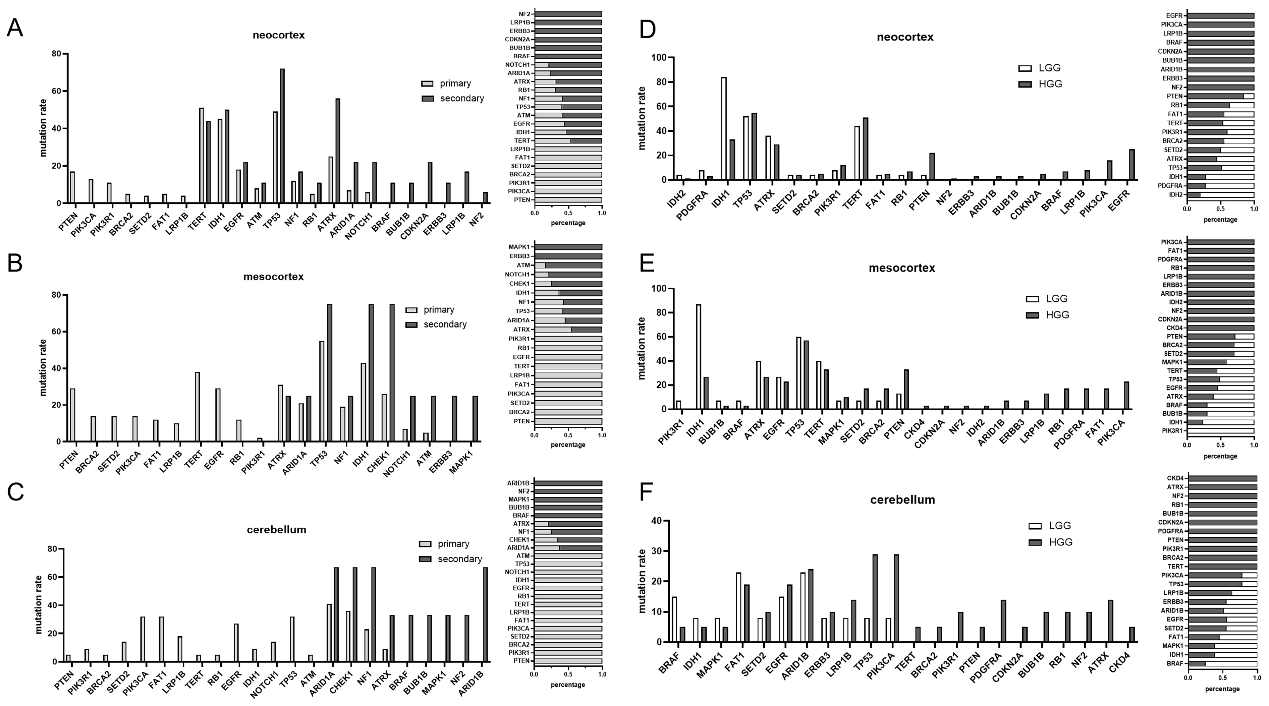


**Fig. S5.** Potential driver genes for recurrence and grade progression of LGG to HGG. Expression of key genes in primary and secondary gliomas of the (A) neocortex, (B) mesocortex, and (C) cerebellum. Expression of key genes in LGGs and HGGs of the (D) neocortex, (E) mesocortex, and (F) cerebellum.

LGG, low-grade glioma; HGG, high-grade glioma.
